# Srcap Chromatin Remodeler Is Required for Efficient Replication Dynamics in Mammalian Cells

**DOI:** 10.3390/ijms262412189

**Published:** 2025-12-18

**Authors:** Stefka K. Dzhokova, Rossitsa H. Hristova, Peter S. Botev, Temenouga N. Guecheva, Anastas G. Gospodinov

**Affiliations:** Roumen Tsanev Institute of Molecular Biology, Bulgarian Academy of Sciences, Acad. G. Bonchev Str. 21, 1113 Sofia, Bulgaria; kirilova0707@gmail.com (S.K.D.); hristova_r@bio21.bas.bg (R.H.H.); petar.botev@gmail.com (P.S.B.); tgesheva@gmail.com (T.N.G.)

**Keywords:** Srcap, chromatin remodeling, DNA replication, replication stress, origin licensing, transcriptional regulation, genome stability

## Abstract

The SNF2-related chromatin remodeler Srcap is the principal ATPase responsible for the deposition of the histone variant H2A.Z at promoters and regulatory chromatin regions. Although this activity is known to modulate transcription, its contribution to DNA replication remains unexplored. Here we show that Srcap is required for efficient replication fork progression and origin firing in mammalian cells. Using RNA interference in human PC3 cells, we found that Srcap depletion leads to a ~25% reduction in fork elongation rate, decreased replication fork density, accumulation of the replication-stress marker γH2AX, and reduced chromatin-bound H2A.Z. High-resolution expansion microscopy further revealed diminished intensity and increased spacing of replication foci, consistent with reduced origin activation. Transcriptomic analysis of published data identified broad downregulation of replication-associated genes. These data uncover a dual mechanism by which Srcap sustains replication efficiency—through both H2A.Z-dependent chromatin organization and transcriptional maintenance of the replication machinery. Our findings establish Srcap as an important coordinator of replication dynamics, with implications for genome stability.

## 1. Introduction

Srcap protein (SNF2-related CREB-binding protein activator protein) is the catalytic core of the SRCAP complex, a large ATP-dependent chromatin remodeling complex with 13 subunits in mammalian cells. The Srcap complex regulates chromatin at promoters and enhancers, influencing gene expression, DNA repair, and chromosome segregation [1,2,3]. Srcap loss or depletion in human and mouse cells reduces H2A.Z incorporation at promoters and other regulatory regions [1,2,4,5,6], leading to widespread misregulation of gene expression [1,4,7]. The need for Srcap for efficient H2A.Z loading has been confirmed by structural and biochemical studies [8,9,10,11,12]. This role of the remodeler is highly conserved across evolution, with homologous complexes such as Swr1 in yeast and p18Hamlet in Drosophila performing analogous functions [1,2,10] and maintaining proper gene-expression programs across diverse developmental and cellular contexts [2,3,4].

Beyond transcriptional control, evidence links Srcap to genome stability. Loss of Srcap results in elevated DNA damage and activation of the replication stress marker γH2AX [13,14]. These phenotypes correlate with a pronounced reduction in H2A.Z on chromatin. On the other hand, the histone variant H2A.Z has been linked to replication origin specification and fork stability. Nucleosomes containing H2A.Z are enriched in H4K20me2 and the origin recognition complex (ORC), defining early replication origins [15,16].

Despite extensive evidence linking SRCAP to transcriptional regulation and H2A.Z deposition, its possible contribution to DNA replication remains insufficiently characterized. Although other chromatin remodelers such as INO80 and SWI/SNF have been implicated in fork stability or replication origin firing [17,18,19,20,21,22], a direct role of SRCAP in these processes has not been established. In particular, it is not known whether SRCAP affects DNA replication primarily through its structural role in H2A.Z incorporation or indirectly via transcriptional regulation of replication-associated genes. On the other hand, understanding how SRCAP contributes to DNA replication is essential for elucidating the chromatin-based mechanisms that preserve genome integrity, with possible implications for various pathologies.

To address this gap, we investigated how SRCAP depletion influences replication dynamics in mammalian cells using a human PC3 cell line depleted of the Srcap core subunit by endonuclease-prepared small interfering RNA (esiRNA). We show that Srcap deficiency is associated with decreases in replication fork elongation rate and fork density. Deficient cells accumulate phosphorylated histone H2AX, indicative of replication stress. This correlates with a significant decrease in the chromatin-bound pool of H2A.Z, suggesting a mechanistic link between defective histone variant deposition and impaired replication origin activity. High-resolution imaging revealed reduced intensity and increased spacing of replication foci in Srcap-deficient cells, consistent with diminished origin firing. Analysis of published RNA-seq data indicated that Srcap deficiency results in severe downregulation in a number of genes required for DNA replication initiation and replication fork stability. Together, these findings show that Srcap loss downregulates replication-gene transcription, decreases origin firing, and slows fork elongation.

## 2. Results

### 2.1. Srcap-Deficient Cells Have Impaired Replication Elongation

**Figure 1:**
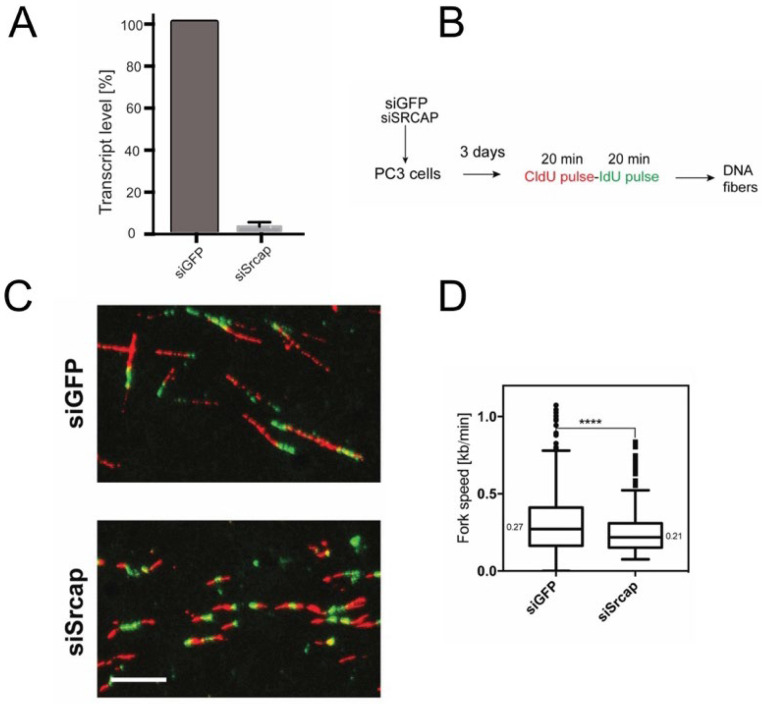
Srcap-depleted cells have impaired DNA replication elongation. (**A**) Quantitative RT-PCR to assess the mRNA levels of Srcap in PC3 cells transfected with esiRNA against GFP (siGFP) or Srcap (siSrcap); mRNA was isolated 3 days after transfection. (**B**) Experimental scheme to measure the rate of DNA replication elongation. (**C**) Control and Srcap-depleted cells, which 3 days after transfection were labeled as in (**B**). Representative images of DNA fibers are shown. Scale bar 10 μm. (**D**) Distribution of fork rates in control (siGFP) and Srcap-deficient cells (siSrcap). Data is from 3 independent experiments; at least 500 fibers were measured per condition. **** *p* < 0.0001. Unpaired two-tailed Student’s *t*-test. Data is shown as a Tukey box plot; numbers indicate the median value.

To study the role of Srcap in replication, we employed RNA interference by endonuclease-prepared small interfering RNA (esiRNA). Since each esiRNA is a complex mixture of many different siRNAs targeting the same mRNA, they cause effective knockdown of target gene expression, while off-target effects are diluted out [23]. Quantitative RT-PCR analysis indicated that three days after the transfection of human PC3 cells, Srcap mRNA levels were strongly reduced in Srcap-depleted cells compared to control cells (Figure 1A).

If Srcap promotes fork progression, its depletion should reduce elongation rates. We measured the rate of elongation in control (transfected with esiRNA against GFP) and Srcap-silenced cells by fiber labeling. Control and Srcap-deficient cells were labeled first with CldU (25 μM for 20 min) and then with 250 μM IdU for 20 min (Figure 1B). After spreading and immunostaining, the lengths of green (second label) tracks of red-green fibers were measured to estimate the rate of movement of forks that were already active when the second label was added. Speed conversion was carried out using a conversion factor of 2.59 kb per µm. The formula used was speed = (length × 2.59)/time according to Jackson and Pombo [24].

Data indicated that the elongation rate in Srcap-depleted cells is reduced (Figure 1C,D) by about 25%. The reduction in the fork elongation rate in Srcap-deficient cells suggests that they experience replication stress.

### 2.2. Srcap Knockdown Induces Accumulation of the Replication Stress Marker Phosphorylated Histone H2AX

The slower forks suggested replication stress, which we next assessed by monitoring γH2AX. Histone variant H2AX is a major substrate of PI3 kinases—ATM [25] and DNA-PK [26]—after double-strand break induction and ATR in response to replication stress [27]. Accordingly, γH2AX is widely used as a highly sensitive biomarker of DNA damage and stress-induced chromatin responses, including replication stress and senescence [28,29].

**Figure 2:**
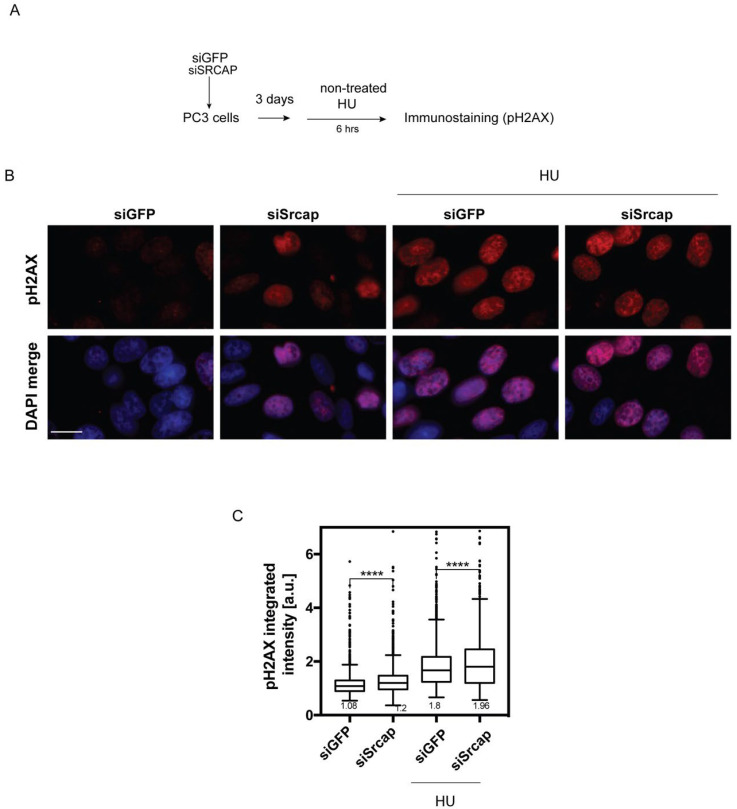
Srcap-deficient cells accumulate phosphorylated H2AX. (**A**) Control and Srcap-silenced PC3 cells were treated 72 h post-transfection with 1 mM HU for 6 h or left untreated. (**B**) Cells were fixed and immunofluorescently stained with an antibody against pH2AX (Ser129). Representative images are shown. Scale bar 10 μm. (**C**) Nuclear staining intensities were analyzed using CellProfiler 4.2. The plot represents the distribution of pH2AX nuclear intensities for each condition. Data is from 3 independent experiments; at least 1200 cells were analyzed per condition. **** *p* < 0.0001. Unpaired two-tailed Student’s *t*-test. Data is shown as a Tukey box plot; numbers indicate the median value.

Staining of Srcap-depleted cells with an antibody against γH2AX indicated an increase in histone H2AX phosphorylation compared to controls (Figure 2B,C, first two columns) confirming the replication stress in these cells. Application of the common replication inhibitor hydroxyurea (HU) further increased the H2AX phosphorylation level, with that in Srcap-deficient cells remaining significantly higher (Figure 2B,C, last two columns). Taken together, the data from fiber-labeling experiments and γH2AX immunostaining indicate that a Srcap deficit causes replication stress.

### 2.3. Srcap-Deficient Cells Have Reduced Amounts of Histone Variant H2A.Z

**Figure 3:**
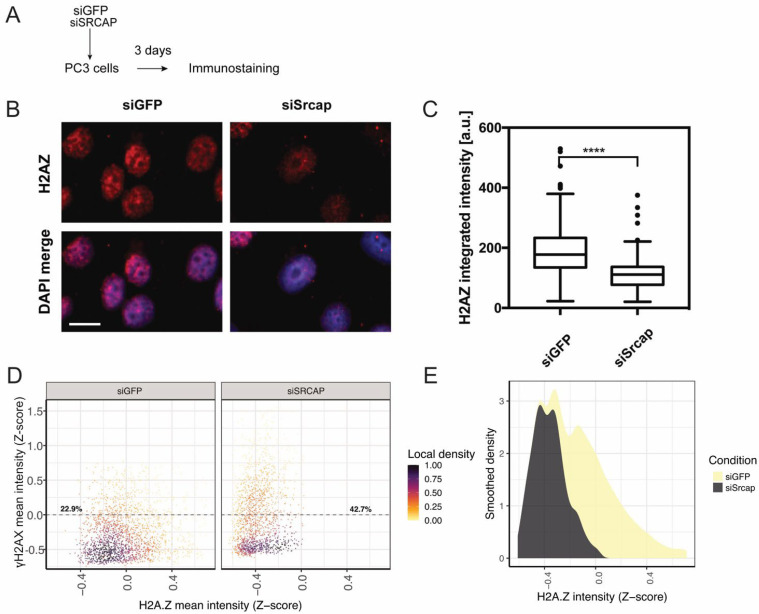
Srcap-deficient cells have reduced H2A.Z on chromatin and experience replication stress. (**A**) Experimental scheme. (**B**) Control and Srcap-silenced PC3 cells, which, 72 h post-transfection, were fixed and immunofluorescently stained with an antibody against H2A.Z. Representative images are shown. Scale bar 10 μm. (**C**) Distribution of H2A.Z nuclear intensities for cells in each condition. Data is from 2 independent experiments; at least 500 cells were analyzed per condition. **** *p* < 0.0001. Unpaired two-tailed Student’s *t*-test. Data is shown as a Tukey box plot. (**D**) Scatter plots of H2A.Z vs. γH2AX intensity in control and Srcap-deficient cells. (**E**) Distribution of H2A.Z intensity among γH2AX-positive cells (defined as cells with nuclear γH2AX intensity above the mean).

The Srcap chromatin remodeling complex has been shown to catalyze the exchange of histone H2A for H2A.Z. To check if Srcap depletion changed the abundance of histone H2A.Z on chromatin, we carried out immunostaining for the histone variant in control and Srcap-silenced cells (Figure 3A) following pre-extraction of soluble proteins. The result (Figure 3B,C) indicated that Srcap-depleted cells displayed a strong (~40%) reduction in nuclear staining intensity for histone H2A.Z, which might contribute to the replication defect in Srcap-depleted cells. To further check the assumption, we co-stained control and Srcap-depleted cells with antibodies against H2A.Z and γH2AX. The data indicated that while siSrcap sample cells were shifted to the left, the amount of γH2AX-positive cells (defined as cells with γH2AX nuclear intensities above the mean) was almost twofold higher (Figure 3D). This population also had a pronounced reduction in H2A.Z nuclear signal (Figure 3E), implying that it might be linked to the replication stress in Srcap-depleted cells.

### 2.4. Srcap-Silenced Cells Have Reduced Replication Fork Density

A recent study in HeLa cells has shown that nucleosomes containing the histone variant H2A.Z may be necessary for replication initiation. Nucleosomes that contain H2A.Z bind the histone lysine methyltransferase enzyme SUV420H1, promoting H4K20me2 deposition, which, in turn, is required for ORC1 binding. H2A.Z-regulated replication origins exhibit higher firing efficiency and earlier replication timing than other origins [30]. These data imply that a reduction in origin firing efficiency in H2A.Z-defective cells would result in fewer forks per unit length of DNA.

**Figure 4:**
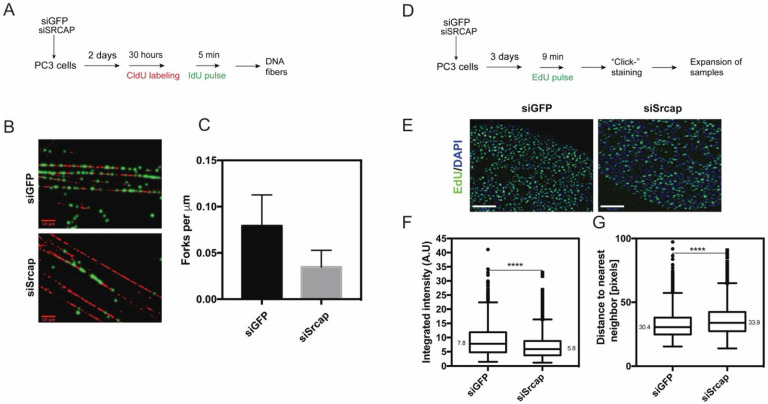
Srcap-depleted cells have reduced replication fork density. (**A**) Experimental scheme to analyze the density of replication forks. (**B**) Representative images of DNA fibers from cells processed as in (**A**) are shown. Scale bar 10 μm. (**C**) Fork density in control and Srcap-deficient cells. Data is from 3 independent experiments, with at least 100 green spots (forks) measured per experiment. (**D**) PC3 cells transfected with esiRNA against GFP and Srcap, which, 3 days after transfection, were pulse labeled with EdU, fixed, and stained. (**E**) Stained cells were processed for expansion microscopy and imaged. Representative images of fragments of nuclei (fitting in the field of view) are shown. Scale bar 10 μm. The staining intensity of individual replication centers (**F**) and the distance to the nearest neighbor (**G**) were measured using CellProfiler. Data is from at least 15 individual cells per condition. At least 1000 replication centers were analyzed in both (**F**,**G**); **** *p* < 0.0001. Unpaired two-tailed Student’s *t*-test. Data is shown as a Tukey box plot; numbers indicate the median value. Consistent results were obtained in two independent experiments.

As we have observed a reduction in H2A.Z nuclear intensity (Figure 3B,C) in Srcap-silenced cells, and these cells have defective replication (Figure 1), it could be expected that the number of active replication forks in these cells is reduced. To assess the number of active replication forks in control and Srcap-depleted cells, they were labeled with CldU for 30 h (time that exceeds the duration of a single cell cycle in PC3 cells) to counterstain total DNA, and the second label (IdU) was applied as a brief pulse (5 min). Fiber labeling using this labeling scheme results in active forks appearing as green dots on the red fiber background (Figure 4A). The result indicated that in Srcap-deficient cells, there was a twofold reduction in replication fork density (Figure 4B,C).

To further elucidate the effect of Srcap knockdown on the organization of DNA replication, we wanted to image replication foci with the highest possible resolution. Image resolution in conventional microscopy cannot exceed half of the wavelength (Abbe limit). In the last decade, various approaches have been developed that make it possible to exceed the Abbe limit; however, this requires dedicated instruments or the use of specific fluorophores. Recently, it has been shown that a preserved biological specimen could be physically magnified if included in a dense cross-linked network of swellable polyelectrolyte hydrogel, thus isotropically expanding biomolecules or labels [31]. Most protocols allow expansion of a sample by about 100× in volume or ~4.5× in the linear dimension. Thus, a microscope with a diffraction limit of ~300 nm would attain an effective resolution of ~300 nm/4.5 ≈ 70 nm. We applied expansion to control and Srcap-depleted cells that were labeled by a short pulse of EdU (Figure 4D). Analysis of images (Figure 4E) using CellProfiler software 4.2 [32] indicated that the intensity of replication foci in Srcap-depleted cells was reduced by about 25% (Figure 4F). At the same time, the distance to the nearest neighbor was increased by about 12% (Figure 4G). Data from fiber-labeling experiments and expansion microscopy (Figure 4) indicate that in Srcap-depleted cells, the density of replication forks is decreased. This decrease is consistent with, and may partly result from, the reduced amount of H2A.Z on chromatin in these cells.

### 2.5. Transcriptomic Analysis of Srcap-Depleted Cells Reveals Replication- and Checkpoint-Related Transcriptional Reprogramming

To explore the molecular pathways affected by Srcap depletion, we reanalyzed data published by Droll et al., 2025 [33]. Because publicly available transcriptomic data for Srcap depletion in PC3 are not yet available, we analyzed this from differentiated human keratinocytes to identify Srcap-dependent gene expression programs. The replication-related transcriptional changes are expected to reflect general consequences of Srcap deficiency, given the conserved chromatin functions of Srcap.

**Figure 5:**
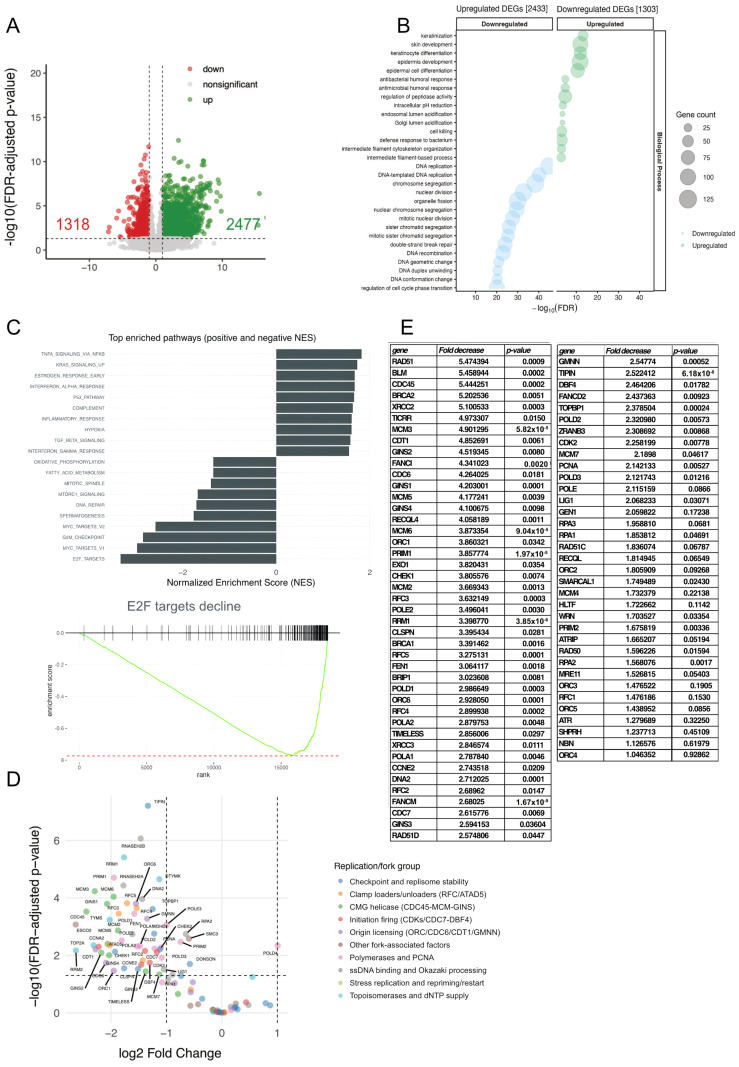
Differential expression analysis of cells depleted of Srcap. (**A**) Volcano plot of differentially expressed genes (DEGs) in Srcap-depleted human keratinocytes relative to controls. Genes with adjusted *p* < 0.05 are shown in color; replication-related factors are highlighted. (**B**) Gene Ontology (GO) over-representation analysis of downregulated DEGs. The top enriched Biological Process terms include “DNA replication,” “replication fork processing,” and “cell-cycle checkpoint signaling.” (**C**) Gene Set Enrichment Analysis (GSEA) using the Hallmark collection identifies suppression of E2F- and Myc-regulated transcriptional programs that drive S-phase entry and replication. The green curve represents the running enrichment score across the ranked gene list, while the red dashed line indicates the maximal (negative) enrichment score. (**D**) Scatterplot illustrating the expression of replication-associated genes grouped by functional categories: initiation, origin licensing, CMG helicase, polymerases/PCNA, and fork-stability factors. (**E**) Quantitative summary showing that ~32% of initiation and ~42% of elongation (polymerase/PCNA) genes are significantly downregulated (adjusted *p* < 0.05), with an average log_2_ fold change of −1.6 (≈0.34×) and −1.4 (≈0.40×), respectively.

Differential expression analysis identified 3795 genes showing significant changes (adjusted *p* < 0.05), with 2477 upregulated and 1318 downregulated (Figure 5A). Gene Ontology (GO) enrichment analysis of differentially expressed genes (DEGs) revealed upregulation of immune and stress response categories, while downregulated genes were strongly enriched for replication-associated processes such as “DNA replication,” “replication fork processing,” “checkpoint signaling,” and “cell cycle regulation” (Figure 5B).

Gene Set Enrichment Analysis (GSEA) using the Hallmark collection confirmed suppression of E2F-dependent transcriptional programs and other replication-linked pathways (Figure 5C), in line with the reduced replication fork activity and checkpoint activation observed experimentally (Figure 1, Figure 2, Figure 3 and Figure 4). Several components of the replisome, including MCM3–7, POLA1, POLE, PCNA, RFC2/4, CDC45, and GINS2, were downregulated, together with CDK2 and DBF4 that drive initiation (Figure 5D–E).

These data establish a transcriptional signature consistent with the reduced fork density and elongation rates observed upon Srcap depletion, suggesting that chromatin remodeling by Srcap may regulate the expression of replication machinery components.

## 3. Discussion

Chromatin regulators are involved in the replicative process both directly and indirectly. Thus, histone post-translational modifications change dynamically relative to replication fork passage [34]. Components of the NuA4 and SAGA complexes; histone chaperones Asf1, FACT, and Nap1; and chromatin remodelers INO80, RSC, and ISW1A are required for efficient replication [35]. We have found that a sister complex to Srcap, the mammalian INO80 complex, is required for efficient replication [18] by suppressing co-transcriptional R-loops [22]. Here, we report that mammalian Srcap contributes to efficient replication, as its depletion is accompanied by reduced replication fork rates and density. This is evidenced by reduced replication fork speeds (Figure 1) and the concomitant accumulation of the replication stress marker phosphorylated histone H2AX (Figure 2)

Two non-exclusive mechanisms likely underlie the phenotype: (i) a chromatin structural route, where diminished H2A.Z impairs origin licensing and firing and (ii) a transcriptional route, where downregulation of replication genes limits initiation capacity and elongation support.

The yeast homolog of the Srcap–SWR complex mediates incorporation of the only histone variant Htz1 (human H2A.Z) into chromatin by exchanging chromatin-bound H2A–H2B dimers with Htz1-H2B dimers [36,37,38]. In mammalian cells, both Srcap and P400 chromatin complexes appear to be able to incorporate H2A.Z into chromatin, possibly with different efficiency and specificity [39].

The observed reduction in H2A.Z levels may contribute to the replication defects seen in Srcap-deficient cells, since a study has demonstrated that genome-wide distribution of ORC1 and nascent DNA strands co-localize with H2A.Z. H2A.Z-containing nucleosomes were found to bind directly to the histone lysine methyltransferase enzyme SUV420H1, promoting H4K20me2 deposition, which is in turn required for ORC1 binding. Thus, H2A.Z epigenetically regulates the licensing and activation of replication origins [30]. In line with this study, we observe a reduction in replication fork density (Figure 4) in Srcap-depleted cells (that also display a reduction in H2A.Z abundance—Figure 3). Reduced fork density in Srcap-deficient cells likely causes their replication centers to appear dimmer and to be at a greater distance from each other when imaged by expansion microscopy (Figure 4). However, although Srcap-depleted cells display both reduced levels of nuclear H2A.Z signal following extraction, as well as impaired replication dynamics, our data is only correlative and does not show direct causation. Therefore, we cannot exclude the possibility that other functions of Srcap—such as broader transcriptional reprogramming or effects on chromatin architecture—contribute independently to the replication defects observed.

Simultaneously, the transcriptional signature of Srcap-knockdown keratinocytes indicates that more than one-third of genes involved in replication initiation and elongation are downregulated, with an average reduction of about 40%. This points to a dominant regulatory effect at the level of gene expression and suggests that transcriptional regulation by the remodeler (Figure 5) is the major cause of the observed replication impairment. Our transcriptome analysis indicates that Srcap depletion leads to coordinated downregulation of multiple genes required for replication initiation and fork progression, including MCM components, polymerases, and checkpoint factors (Figure 5). Although the precise mechanism remains to be fully elucidated, several chromatin-based routes may explain how Srcap regulates these transcriptional programs. First, Srcap-mediated H2A.Z deposition is enriched at promoters and enhancers, where H2A.Z-containing nucleosomes facilitate an open chromatin state conducive to transcriptional initiation [1,40]. Consequently, loss of H2A.Z may reduce promoter accessibility or alter the binding of transcription factors. Second, Srcap could influence histone acetylation patterns based on its interactions [1,41] and could conceivably modulate promoter competence and transcriptional amplitude. Finally, impaired chromatin organization due to reduced H2A.Z incorporation could affect the recruitment of transcriptional regulators, leading to attenuation of replication-related gene expression. Future work dissecting each of these mechanisms will be required to determine precisely how Srcap-dependent chromatin remodeling drives the observed transcriptional changes.

An important challenge for the future would be to understand how the replication defect phenotype in Srcap-deficient cells affects people with Srcap-related syndromes such as Floating-Harbor syndrome [42].

## 4. Materials and Methods

### 4.1. Cell Culture and RNA Interference

Human PC3 cells were grown in DMEM supplemented with 10% fetal serum, 1 mM pyruvate, and antibiotics in a 5% CO_2_ atmosphere.

EsiRNAs targeting the coding regions of human Srcap (663-1073, transcript NM_006662.1) or GFP (132-591) were synthesized as previously described [23,43]. Primers used to amplify the targeted regions were selected using the Riddle database [44].

Quantities of Lipofectamine and esiRNAs for efficient knockdown were optimized using esiRNA against Eg5 (Kif11). Typically, 60 pmol of esiRNA and 2 μL of Lipofectamine 2000 (Invitrogen, Carlsbad, CA, USA) were used per well in a 24-well plate (500 mL transfection volume). Knockdown was assessed by quantitative RT-PCR. Co-transfections of esiRNAs and plasmids were carried out as above with 45 pmol esiRNA and 200 ng plasmid DNA.

### 4.2. DNA Fiber Labeling

DNA fiber analyses were performed as described by Schwab and Niedzwiedz [45] with slight modifications. Briefly, exponentially growing PC3 cells were first incubated with 25 mM chlorodeoxyuridine (CldU) and then with 250 mM iododeoxyuridine (IdU) for the indicated times. (For inter-origin distance measurements, labeling was first with IdU at 25 mM and then with CldU at 250 mM). Spreads were prepared from 2500 cells (suspended in PBS at 1 × 10^6^ cells/mL). Cell lysis was carried out in fiber lysis solution (50 mM EDTA and 0.5% SDS in 200 mM Tris-HCl, pH 7.5). DNA fibers were spread by tilting the slides ~25 degrees until the drop of the fiber solution reached the bottom of the slide and was allowed to dry. Dried slides were either stored at 4 °C or processed immediately. Slides were suspended in 2.5 M HCl for 80 min, washed in PBS, and then incubated in blocking buffer (5% bovine serum albumin in PBS) for 40 min. Primary antibodies—mouse anti-BrdU antibody (Becton Dickinson, cat # 347580) to detect IdU and rat anti-BrdU antibody (Abcam cat# Ab6326) to detect CldU—were diluted in blocking buffer and applied overnight. Slides were washed several times in PBS, incubated with secondary antibodies (anti-rat DyLight 594 and anti-mouse DyLight 488, Abcam, Cambridge, UK) for 60 min, and mounted with ProLong Gold anti-fade reagent (Molecular Probes, Eugene, OR, USA). Images were acquired with an Axiovert 200M microscope (Carl Zeiss, Oberkochen, Germany) equipped with an Axiocam MR3 camera (Carl Zeiss). Fiber length measurements were carried out using AxioVision 4.7 software (Carl Zeiss), Image J (version 2.16), or DNA size finder [46]. For replication rate estimations, the lengths of the IdU-labeled (green) tracts within red-green tracks were measured. Fork speed was calculated using a conversion factor of 2.59 kb per μm, applying the formula speed = (track length × 2.59)/labeling time, as described by Jackson and Pombo [24]. For fork-density measurements, CldU was applied for 30 h to uniformly label DNA synthesized during the preceding cell cycle, allowing visualization of the full length of individual DNA fibers. A brief 5 min IdU pulse selectively marked active replication forks at the time of collection, enabling quantification of fork density per unit length of pre-labeled DNA.

### 4.3. Immunofluorescence

For immunofluorescence, cells were grown on coverslips, washed in PBS, fixed with ice-cold methanol for 7 min at −20 °C, permeabilized with 0.5% Triton X-100 in PBS for 5 min, washed with PBS, and blocked in 5% bovine serum albumin (BSA) in PBS containing 0.05% Tween (PBS-T) for 1 h. Staining was performed using either mouse anti-phospho H2AX(S129) antibody (BioLegend, San Diego, CA, USA) diluted 1:200 or rabbit anti-H2A.Z antibody (Abcam) using the same dilution overnight at 4 degrees C. Slides were then washed 3 × 5 min in PBS-T, and secondary IgG DyLight 594 goat anti-rabbit and anti-mouse antibodies were used at 1:500 dilution for 1 h at room temperature. For H2A.Z staining before fixation, cells were pre-extracted with CSK buffer (Triton X-100, 100 Mm NaCl, 3 mM MgCl_2_, 300 mM sucrose, 1 mM EGTA, and 10 mM HEPES pH6.8) containing protease inhibitors (Complete^TM^ inhibitor cocktail, Roche, Basel, Switzerland) and phosphatase inhibitors (Sigma, Kawasaki, Japan) and incubated for 10 min on ice. Microscopy was carried out using an Axiovert 200M wide-field microscope (Carl Zeiss). For the analysis of replication foci in expansion microscopy datasets, EdU-positive foci were identified as individual objects. For each focus, integrated intensity and nearest-neighbor distances were quantified.

### 4.4. Expansion Microscopy

Control and Srcap-depleted cells were pulse-labeled with EdU (25 μM) for 2 min and allowed to incorporate the already internalized precursor for 7 more minutes in a fresh medium. Methanol-fixed cells were incubated with 5% bovine serum albumin in PBS and “click”-stained with Alexafluor 488 azide using a mixture containing 100 mM Tris-HCl, pH = 7.6, 10 mM CuSO_4_, 20 μM dye, and sodium ascorbate 200 mM for 30 min at RT. The addition of anchors was carried out by incubating the coverslips in a mixture (prepared just before use) containing 0.7% formaldehyde and 1% acrylamide at 37 °C for 5 h. Gelation was carried out by placing the coverslip face down on a drop containing 36 microliters monomer solution (23% w/v sodium acrylate, 10% acrylamide, and 0.1% N, N′-methylenbisacrylamide in PBS), 2 microliters 10% TEMED, and 2 microliters 10% APS. To allow sufficient time for the solution to penetrate the cells before gelation components and the humid chamber were cooled;the monomer solution was kept at −20 °C, and TEMED and APS solutions were kept on ice. Mixture drops were placed in a gelation chamber kept for at least 30 min at −20 °C. After 5 min on ice, the coverslips in the gelation mixture were moved to 37 °C for 1 h. Coverslips were placed in digestion buffer (containing 0.5% Triton X-100, 1 mM EDTA, 50 mM Tris-HCl (pH = 8.0), 1 M NaCl, and 8 U/mL proteinase K) at 37 °C overnight. After digestion, gels separated from coverslips were expanded in 250 mL beakers containing distilled water, with three successive water changes at 30 min intervals. Pieces of expanded gels were placed on poly-L-lysine glass-bottom Petri dishes and imaged on an Andor Dragonfly 505 spinning disk confocal system. To further increase resolution, the system was used in SRRF-Stream mode [47,48]. The EdU intensity of each replication center and the distance to the nearest neighbor were measured by CellProfiler software 4.2 [32].

### 4.5. RNA-Seq Analysis and Pathway Enrichment

We analyzed RNA-seq data published by Droll et al., 2025 [33]. Of the dataset (GSE278641) we analyzed the samples corresponding to differentiated control and Srcap knockdown human keratinocytes. Raw sequence data were retrieved from the Sequence Read Archive (SRA) and pseudoaligned to the human transcriptome (GRCh38) using Salmon [49] in selective-alignment mode. Quantification indices were generated per sample, and transcript-level abundance estimates were imported into DESeq2 [50] through the tximport pipeline. Differential expression analysis was conducted with DESeq2 using the Wald test and log_2_ fold change. Genes with adjusted *p* values (padj) < 0.05 were considered significantly differentially expressed.

Functional enrichment analyses were performed in R using the clusterProfiler (v. 4.10.1) [51] and fgsea (version 1.28) [52,53] packages. Over-representation analysis (ORA) for Gene Ontology Biological Process (GO:BP) terms was conducted with Benjamini–Hochberg multiple-testing correction. Gene Set Enrichment Analysis (GSEA) was performed using fgsea with the MSigDB Hallmark gene sets (collection H, version 20vx24.1), ranking genes by log_2_ fold change.

Volcano plots were produced using ggplot2, distinguishing up- and downregulated genes by color and marking significance thresholds for padj and log_2_ fold change.

### 4.6. Statistics

Quantitative imaging data were extracted using CellProfiler and exported for statistical evaluation. All statistical analyses were performed using GraphPad Prism (version 7) and R (version 4.3). For comparisons between two conditions, statistical significance was assessed using unpaired two-tailed Student’s *t*-tests, consistent with established practice for DNA fiber assays and quantitative immunofluorescence measurements. Data are presented as Tukey box plots with medians indicated.

RNA-seq differential expression analysis was carried out with DESeq2 using the Wald test. Multiple-testing correction was performed using the Benjamini–Hochberg procedure, and genes with adjusted *p* < 0.05 were considered significantly differentially expressed. Gene Ontology over-representation and GSEA enrichment analyses were performed using clusterProfiler and fgsea, applying FDR < 0.05 as the significance cutoff. Unless otherwise stated, a significance threshold of *p* < 0.05 (or FDR < 0.05 for RNA-seq-based analyses) was applied throughout.

## Data Availability

The original contributions presented in this study are included in the article. Further inquiries can be directed to the corresponding author.
